# The impact of sports participation on the psychological state of the Chinese population from the perspective of sports resources

**DOI:** 10.1371/journal.pone.0349696

**Published:** 2026-05-19

**Authors:** Bin Li, Xianhong Zeng, Shuyu Ji

**Affiliations:** 1 College of Physical Education, NorthWest Normal University, Lanzhou, China; 2 College of Physical Education and Sport, Beijing Normal University, Beijing, China; 3 College of Sports and Great Health, Yibin University, Yibin, China; West Pomeranian University of Technology, POLAND

## Abstract

**Objective:**

Against the backdrop of the Chinese people’s growing demand for health, it is of great significance to explore the impact of sports participation on different psychological states among the population, as well as the specific mechanisms and theoretical adaptations under varying conditions of sports resources—such as the sports environment, facilities, and safety. Such an investigation not only contributes to the widespread promotion of public participation in sports but also provides targeted theoretical guidance for improving psychological well-being.

**Method:**

Using data from the China General Social Survey (CGSS) published in 2023, a linear regression model was constructed.

**Results:**

Sports participation exerts a comprehensive positive effect on the psychological well-being of the Chinese population. Robustness tests using instrumental variable methods confirm that the impact of sports participation on psychological well-being is free from endogeneity issues and exhibits strong robustness. The influence of sports participation on psychological well-being varies significantly depending on sports resource conditions: ① Under favorable sports environment conditions, it exerts a comprehensive positive effect; under poor sports environment conditions, it has no effect on depression and despondency. ② In well-equipped facilities, it more effectively promotes calmness and vitality, while in under-equipped facilities, it has no effect on depression/despondency or calmness. ③ Under low-safety conditions, it has a comprehensive positive effect, while under high-safety conditions, it has no effect on depression/despondency.

**Conclusion:**

Sports participation exerts a robust, positive influence on overall mental well-being and vitality at the macro level. However, its effects manifest in complex interrelationships under specific sports resource conditions. Recommendations: Comprehensively enhance the role of sports participation in improving public mental health; Implement intelligent strategies for sports resource development to amplify the positive impact of sports participation on psychological well-being.

## 1. Introduction

The psychological well-being of the Chinese population and their relatively low levels of sports participation have become key research concerns in the fields of mental health and public health [1]. It is now well-established that engaging in sports serves as an effective, evidence-based intervention for a range of mental health conditions [2]. Regular, structured participation in physical activity has been shown to reduce inflammatory markers in the body, thereby contributing to improved mental health outcomes [3]. Numerous scholars have investigated this topic from physiological and biochemical perspectives, elucidating the mechanisms by which endorphins, mitochondria, mammalian target of rapamycin (mTOR), neurotransmitters, and the hypothalamic-pituitary-adrenal (HPA) axis contribute to mental health improvements and enhanced psychological well-being through sports participation [4]. These experimental findings support the assertion that sports participation regulates internal physiological and biochemical processes to alleviate negative psychological states and promote positive ones, offering robust evidence for its effectiveness in improving mental well-being. However, not all research concurs. Some scholars have reported that exercise interventions do not significantly improve certain negative mental health conditions [5]. From a sociological perspective, sports participation has been found to enhance positive psychological dimensions such as social identity and community belonging [6]. Consequently, the relationship between sports participation and mental health remains marked by several unresolved issues: ① Existing studies yield inconsistent or even contradictory findings regarding the effectiveness of physical activity in addressing mental health concerns. It remains unclear whether sports participation universally benefits mental health, or whether its effects are limited to specific psychological conditions. ② Variability in the indicators used to assess psychological well-being may contribute to divergent results. Experimental research often relies on objective physiological measures, such as near-infrared spectroscopy and electroencephalography (EEG), to examine the effects of physical activity on brain function. In contrast, sociological studies typically employ subjective tools, such as self-reported scales and questionnaires, which reflect participants’ perceptions and intentions. This methodological divergence may account for the inconsistencies observed. ③ Experimental conditions in physiological and biochemical studies—such as specific exercise types, intensity, duration, and controlled settings—are difficult to replicate in real-life contexts. These designs minimize individual variability and isolate the effects of exercise, but they also limit the generalizability of findings to broader populations. When experimental controls are removed and individuals return to their daily routines, it remains uncertain whether sports participation continues to exert a beneficial influence on mental health. This gap is rarely addressed in existing literature. Therefore, how to effectively and broadly harness sports participation to promote mental well-being in real-world settings has emerged as an urgent and underexplored research question.

Psychological states encompass not only a range of negative mental health conditions—such as anxiety, depression, and bipolar disorder [7]—but also a spectrum of positive and optimistic mental states, including optimism, courage, and vitality [8]. Within the general population, positive, neutral, and negative psychological states coexist in varying proportions and significantly influence individuals’ daily experiences and overall quality of life [9,10]. Accordingly, this study adopts vitality as an indicator of a positive psychological state, calmness as a representation of emotional stability, and depression and despair as indicators of negative psychological states. Based on the social nature of human behavior, individuals’ subjective attitudes toward these three dimensions are used to reflect their overall psychological condition. The primary objective of this study is to explore which specific psychological states are significantly affected—or unaffected—by participation in sports. Distinct from previous research constrained by experimental designs, this study removes rigid controls regarding the type, intensity, or duration of physical activity. Instead, it focuses solely on the frequency of sports participation, capturing how engagement in sports manifests in individuals’ personalized and socially embedded daily lives. Moreover, as Chinese society transitions from rapid growth to high-quality development, many regions continue to experience imbalanced and inadequate development. These disparities result in unequal access to sports resources across different geographic areas. Variations in sports environments, available facilities, and safety conditions can influence both the frequency of participation in sports and its psychological impact. In response, this study addresses the research question: What is the impact of sports participation on the psychological states of Chinese citizens from the perspective of sports resources? To answer this, the study utilizes data from the China General Social Survey (CGSS) and constructs a statistical model to examine the causal relationship between sports participation and multiple dimensions of psychological states among the Chinese population. This study examines the effects of sports participation on different psychological states among the Chinese population and investigates the underlying mechanisms under varying conditions of sports resources—including the sports environment, facilities, and safety—through a systematic theoretical analysis. Through an integrative approach combining theoretical derivation and empirical testing, the study seeks to establish a robust theoretical framework explaining how sports participation promotes psychological well-being, and how sports resources mediate or moderate this relationship. This contributes to expanding the explanatory scope and precision of existing theories on sports and mental health. Grounded in statistical evidence and the practical realities of Chinese society, the study offers actionable insights into enhancing the psychological well-being of the population through increased sports engagement.

## 2. Literature review

### 2.1. The impact of sports participation on the psychological state of the Chinese people

As previously discussed, the impact of sports participation on psychological well-being remains a topic of considerable academic debate. Three main dimensions of this debate pertain to the effects of physical activity on positive psychological states, stable psychological states, and negative psychological conditions. Within each dimension, scholars have put forth competing theoretical perspectives, leading to mixed and often contradictory conclusions.

(1) The impact of sports participation on positive psychological states is particularly contested. ① The perspective is grounded in social cognitive theory. It highlights that individual participation in physical activities aligns with the triadic interaction of cognition, behavior, and environment, serving as a key manifestation of the “triadic interaction theory” within social cognitive theory [11]. During the interaction among these three elements, a state of “flow” emerges, enabling participants to experience pleasure and a sense of mastery. This, in turn, stimulates self-efficacy and boosts self-confidence, energizing participants and enhancing their positive psychological state [12]. ②The suppression hypothesis draws on flow interruption theory. It posits that negative experiences during physical activity—such as fatigue, muscle soreness, and injuries—forcefully interrupt the release of endorphins and dopamine in the human body, creating a psychological adaptation gap [13]. This disruption blocks the regulatory effect of physical participation on positive psychological states (e.g., feeling energized) [14]. Due to the existence of opposing viewpoints, Hypothesis H1 is proposed to examine the impact of sports participation on the positive psychological state of the Chinese people and the applicability of the theoretical framework.
(2) The impact of sports participation on emotional stability (i.e., a stable psychological state) is also interpreted through divergent physiological and biochemical frameworks. ① The promotion perspective is grounded in neurotransmitter regulation theory. It posits that physical activity stimulates the release of neurotransmitters such as endorphins and serotonin, thereby reducing anxiety and enhancing emotional stability. This, in turn, helps participants maintain a calm and balanced psychological state [15]. ② The suppression hypothesis draws on the overstress theory. It posits that exercise exceeding the body’s capacity activates the hypothalamic-pituitary-adrenal axis, releasing cortisol and triggering chronic stress responses. This inhibits the release of neurotransmitters such as endorphins and serotonin, leading to emotional fluctuations and disrupting psychological equilibrium [16,17]. Given the opposing viewpoints, Hypothesis H2 is proposed to examine the impact of sports participation on the psychological stability of the Chinese people and to test the applicability of the theoretical framework.
(3) The primary perspective on physical activity participation addressing negative mental health issues emphasizes improvement. It suggests that moderate physical activity enhances the regulatory function of the hypothalamic-pituitary-adrenal axis, reduces stress hormone levels such as cortisol, and simultaneously releases neurotransmitters like endorphins and serotonin that stimulate happiness. By improving participants’ physiological functions, it regulates negative mental health issues like depression and despondency [17]. To further examine this theoretical perspective and its applicability to negative mental health issues among the Chinese population, Hypothesis H3 is proposed.

H1: Sports participation enhances positive psychological well-being among the Chinese population.

H2: Sports participation enhances psychological stability among the Chinese population.

H3: Sports participation mitigates negative psychological health issues among the Chinese population.

### 2.2. The impact of sports participation on the psychological state of the Chinese people under conditions of limited sports resources

As Chinese society shifts from rapid development to high-quality development, it faces pressing challenges related to the uneven distribution of sports resources and an underdeveloped support system. These limitations constrain the capacity of the population to engage in physical exercise as a means of improving psychological well-being. The availability and quality of sports environments, facilities, and safety infrastructure have become key factors influencing both sports participation and its mental health outcomes. Theoretical perspectives on these factors reveal significant divergences, which merit closer examination.

(1) Sports Environment. ① The theory of ecological sports posits that natural environments and sports participation form a symbiotic relationship. Through the connecting effect of natural environments, it enhances participants’ enjoyment of exercise, fostering sustainable motivation for sports engagement. This ensures participants derive pleasure at the subjective level and receive physiological stimulation from endorphins, dopamine, and other neurotransmitters, thereby achieving long-term improvements in psychological well-being [18]. ② The Built Environment Deficiencies Theory posits that deficiencies in urban construction create physical barriers to sports participation among the Chinese population, thereby limiting or obstructing the motivation and behavior associated with engaging in physical activity. Examples include sports parks lacking shade structures, insufficient seating, mixed-use cycling and walking paths, and low per capita sports facility coverage (only 1.5 square meters per person in 2020) [19]. Due to the existence of opposing viewpoints, Hypothesis H4 is proposed to further validate the impact of sports participation on the Chinese population and the applicable theories under different exercise environments.
(2) Sports Facilities. ① Social support theory refers to the provision of social assistance through material or psychological means. By opening school sports facilities to the community, social assistance promotes shared use of athletic resources, builds social networks among citizens, and enhances participation consistency in physical activities. Mixed-age sports participation strengthens social motivation, cultural identity, and group cohesion, thereby effectively improving psychological well-being [20]. ② The theory of resource misallocation indicates that although per capita sports facility space has gradually increased, its distribution remains disconnected from population residential patterns. This results in issues such as a severe shortage of facilities in urban core residential areas and widespread underutilization of sports infrastructure in suburban areas. This structural contradiction of resource misallocation forces regular participants in urban core residential areas to reduce their exercise frequency, while potential participants are compelled to abandon their exercise plans, ultimately impacting psychological well-being [21]. Due to the existence of opposing viewpoints, Hypothesis H5 is proposed to further validate the impact of sports participation on the Chinese population and the applicable theories under different sports facility conditions.
(3) Sports Safety. ① Environmental safety assessment theory indicates that the absence of specialized protective equipment and emergency response measures for specific sports activities, coupled with the lack of regular maintenance for commonly used sports equipment, significantly increases the likelihood of sports injuries and safety incidents. This, in turn, inhibits improvements in psychological well-being [22]. ②Health Belief Theory suggests that comprehensive safety measures can significantly reduce exercise risks and promote stable improvements in psychological well-being [23]. Due to the existence of opposing viewpoints, Hypothesis H6 is proposed to further validate the impact of sports participation on the Chinese population and the applicable theories under different exercise safety conditions.

H4: The impact of sports participation on the psychological well-being of the Chinese population varies according to the quality of the sports environment.

H5: The impact of sports participation on the psychological well-being of the Chinese population varies according to the adequacy of sports facilities.

H6: The impact of sports participation on the psychological well-being of the Chinese population varies according to the level of sports safety.

## 3. Research design

### 3.1. Data sources and sample selection

The data for this study were sourced from the China General Social Survey (CGSS) project, administered by Renmin University of China. The dataset was released in 2023 on the official website of the China National Survey Database (CNSDA) (https://www.cnsda.org/index.php?r=projects/view&id=65635422). This project conducted extensive sampling across China, covering all 31 provinces. Prior to implementation, the project obtained ethical approval. During data collection, informed consent forms were presented before anonymous data was obtained. Designed to provide researchers with comprehensive, authoritative, and scientific data support, subsequent researchers are exempt from seeking ethical approval or presenting informed consent forms. Following data cleaning as required by the study design, a total of 2,577 valid samples remained.

Ethics declaration: Our research was conducted in accordance with the Declaration of Helsinki. Since we utilized publicly available databases, we did not obtain separate ethical approval. The China General Social Survey (CGSS) provides corresponding ethical certification and informed consent through Renmin University of China and the China Survey and Data Center. Furthermore, the CGSS explicitly states that it offers free data resources to researchers and the public, thus eliminating the need for separate approval for this study.

### 3.2. Variable operationalization and explanation

(1) Implicit Variable (Psychological State, PS). To measure levels of depression, calmness, and vitality, the CGSS employs a five-point Likert scale with the following coding: “Always = 1; Often = 2; Sometimes = 3; Rarely = 4; Never = 5.” The treatment of these variables in the analysis is as follows: ① Depression is positively coded in the original dataset, so no transformation is necessary. ② For calmness and vitality, the original coding is reverse-scored. To facilitate more intuitive interpretation of regression coefficients—where higher scores reflect better psychological states—the coding is adjusted to a positive direction: “Always = 5; Often = 4; Sometimes = 3; Rarely = 2; Never = 1.” This study aims to assess the subjective perceptions of three distinct psychological states among the Chinese population. As shown in Table 1, the average scores for depression and discouragement, calmness and composure, and vitality all fall between 3 and 4, indicating a generally favorable overall situation consistent with the realities of Chinese society.
(2) Independent Variable (**Sports Participation, SP).** In the CGSS, sports participation is measured using a five-point scale with the following original coding: “Daily = 1; Several times a week = 2; Several times a month = 3; Several times a year = 4; Never = 5,” which represents reverse coding. To enhance the interpretability of regression coefficients—where higher values indicate more frequent participation—this coding was transformed to a forward direction: “Never = 1; Several times a year = 2; Several times a month = 3; Several times a week = 4; Daily = 5.” To assess the frequency of physical exercise among the Chinese population, as shown in Table 1, it is evident that Chinese citizens tend to engage in physical exercise several times per month, reflecting the realities of Chinese society.
(3) Conditional Variable (Sports Resources, SR). Physical activity resources are represented by three variables: sports environment, sports facilities, and sports safety. In the CGSS, these factors are assessed based on conditions within a one-kilometer radius or a 15-minute walking distance from the participant’s residence. The response options are coded as follows: “Very suitable = 1; Suitable = 2; Average = 3; Unsuitable = 4; Very unsuitable = 5,” which constitutes reverse coding. To address the research objectives, these variables were dichotomized into categories of better versus worse conditions. Using the mean value as the threshold, scores above the average were classified as worse/deficient/low (coded as 1), while scores below the average were classified as better/adequate/high (coded as 2). This study aims to assess the current state of sports resources available to the Chinese population for physical participation. As shown in Table 1, the sports environment is relatively poor, sports facilities are adequately provided, and sports safety is at a moderate level.
(4) **Control Variables.** Following the research paradigms of Ji et al.(2024) [24], Shen et al. (2025) [25], and Li et al. (2025) [26], the control variables selected for this study include gender, age, household registration (hukou status), marital status, physical health, social trust, social equity, medical insurance, and pension insurance.

**Table 1:** Descriptive statistics.

Variable dimension	Variable	Number	Coding method	Mean (SD)	Causality
Implicit variable	depression	*A17*	Always = 1; Often = 2; Sometimes = 3; Rarely = 4; Never = 5	3.930(1.071)	+
(PS)	calmness	*E9*	Always = 5; Often = 4; Sometimes = 3; Rarely = 2; Never = 1	3.975(.951)	+
	vitality	*E10*	Always = 5; Often = 4; Sometimes = 3; Rarely = 2; Never = 1	3.670(1.066)	+
Independent Variable	Sports Participation (SP)	*A30_9*	Every day = 5; Several times a week = 4; Several times a month = 3; Several times a year = 2; Never = 1	2.835(1.615)	+
Conditional Variable	sports environment	*E36_A*	Classified by average value; <average value is better = 2, > average value is worse = 1	1.239(.426)	classify
(SR)	sports facilities	*E36_B*	Classified by average value; < average value is relatively complete = 2, > average value is relatively incomplete = 1	1.901(.824)	classify
	sports safety	*E36_C*	Classified by average value; < average value is high = 2, > average value is low = 1	1.517(.499)	classify
Control variable	gender	*A2*	Male = 1; Female = 2	1.544(.498)	classify
(CV)	age	*A3-1*	Based on year of birth	1968.819(17.342)	+
	household registration	*type*	Rural areas = 1; urban areas = 2	1.440(.496)	classify
	marriage	*A69*	Single = 1; Married = 2	1.733(.442)	classify
	good health	*A15*	Continuous variables 1–5	3.466(1.082)	+
	social trust	*A33*	Continuous variables 1–5	3.674(.989)	+
	social equity	*A35*	Continuous variables 1–5	3.459(.974)	+
	health insurance	*A61_1*	Participated = 1; Did not participate = 2	1.058(.234)	classify
	old-age insurance	*A61_2*	Participated = 1; Did not participate = 2	1.263(.440)	classify

Note: Omit the single-digit value if it is 0.

The descriptive statistics of the sample are presented in Table 1. The “Number” column corresponds to the variable codes used in the CGSS dataset, which serve as the symbolic identifiers for the variables in this study.

### 3.3. Method selection and model construction

(1) **Method Selection.** The research question of this study is “The Impact of Sports Participation on the Psychological State of the Chinese People from the Perspective of Sports Resources.” This constitutes a study of the variable relationship between X and Y. Since the dependent variable in this study is an ordered hierarchical variable, the ordered logistic regression model, which is more suitable for this purpose, is selected as the statistical method for this research.
(2) **Model Construction.** To test the hypotheses proposed in this study, guided by the model construction methodologies outlined in Qiu et al. (2018) [27], the following model was constructed:

$${MS}_{y}=\frac{1}{{1+e}^{-\alpha_{0}+\sum {\alpha_{n}CV}_{n}+\varepsilon }}$$ (1)

$${MS}_{y}=\frac{1}{{1+e}^{-\alpha_{0}+\alpha_{1}PS+\sum \alpha_{n}X_{n}+\varepsilon }}$$ (2)

$${MS}_{yj}=\frac{1}{{1+e}^{-\alpha_{0}+\alpha_{1}{PS}_{j}+\sum \alpha_{n}X_{nj}+\varepsilon }}$$ (3)

Formula (1) represents Model 1 (the base model). In this model, *PS* denotes the dependent variable (psychological state), where y refers to the three specific psychological variables: depression and sadness, calmness, and vitality. α is the intercept term and the coefficient of the independent variable. *CV* represents the set of control variables, with *c* indicating an individual control variable, and ε is the random error term. Formula (2) corresponds to Model 2, which incorporates *SP* as the independent variable of sports participation. Formula (3) represents Models 3–8 (conditional models), where *j* denotes the binary conditions associated with the three dimensions of sports resources: sports environment, sports facilities, and sports safety, respectively.

## 4. Results analysis and discussion

### 4.1. The impact of sports participation on the psychological state of the Chinese people

As demonstrated by Model 2 in Table 2, the effect of sports participation on depression and despondency (A17) among the Chinese population is statistically insignificant, with p > 0.05. Observing the R² values, Model 2 (R² = .081) shows only a marginal increase of 0.001 compared to Model 1 (R² = .080) (see Table 2). This indicates that physical activity participation contributes minimally to the psychological state of depression and despondency among the Chinese population. Consequently, no significant correlation exists between physical activity participation and the psychological state of depression and despondency among the Chinese population. Hypothesis H3 is rejected. Several potential explanations for this finding include: ① The correlation between sports participation and negative mental health outcomes is relatively weak. As demonstrated by Zhou et al.(2021) [28], sports participation is not significantly associated with negative mental health issues such as depression and discouragement, and even individuals with frequent sports engagement—such as university students—are not necessarily protected from these conditions. ② The effectiveness of sports participation alone in improving mental health is limited. According to Johnston et al.(2021) [29], while sports participation may exert some positive effects on sleep disturbances linked to negative mental health, relying solely on sports participation to ameliorate depressive and discouraged psychological states is insufficient.

**Table 2:** Results of the impact of sports participation on the psychological state of the Chinese people.

Variable	1: basic model			2: Participation in sports		
	A17	E9	E10	A17	E9	E10
A30_9				.044(.024)	.115(.024)***	.213(.023)***
A2	−.333(.074)***	−.226(.074)**	−.182(.072)*	−.324(.074)***	−.205(.074)**	−.145(.073)*
A3-1	−.006(.002)**	−.007(.002)**	.004(.002)	−.007(.002)**	−.008(.002)***	.003(.002)
type	−.283(.075)***	−.385(.075)***	−.324(.074)***	−.251(.077)***	−.302(.077)***	−.177(.076)*
A69	.326(.085)***	−.006(.085)	.161(.083)	.328(.085)***	.002(.085)	.174(.084)*
A15	.815(.040)***	.464(.038)***	.722(.039)***	.810(.040)***	.453(.038)***	.707(.039)***
A33	.093(.040)*	.155(.040)***	.134(.039)***	.090(.040)*	.147(.040)***	.117(.039)**
A35	.129(.040)***	.117(.040)**	.047(.040)	.126(.040)**	.110(.041)**	.038(.040)
A61_1	.059(.167)	.066(.163)	−.031(.162)	.065(.167)	.073(.163)	−.022(.163)
A61_2	−.019(.089)	−.042(.089)	.151(.088)	−.015(.089)	−.028(.089)	.177(.089)*
cut1	−14.703(4.823)	−17.708(4.776)	7.102(4.678)	−14.986(4.828)	−18.768(4.795)	5.933(4.708)
N	2577	2577	2577	2577	2577	2577
R^2^	.080	.038	.070	.081	.041	.082

Note: (1) * indicates P < 0.05, ** indicates P < 0.01, and *** indicates P < 0.001; (2) The values in parentheses are standard errors.

The effects of sports participation on calmness (E9) and vitality (E10) are both statistically significant at the p < 0.001 level. Examining the R² values, compared to Model 1, calmness increased from 0.038 to 0.041 (approximately a 8% increase), and vitality increased from 0.070 to 0.082 (approximately a 17% increase). This indicates that sports participation exhibits a strong positive correlation with the Chinese people’s calm and stable psychological state and their energetic and positive psychological state. Hypotheses H1 and H2 are thus validated. Several potential explanations for this finding include: ① Sports participation stimulates the release of “happiness factors.” Physical activity enhances physiological mechanisms that improve brain function and emotional well-being by stimulating neurotransmitters such as dopamine, endorphins, and serotonin. This, in turn, promotes a calm and balanced psychological state and elevates an energetic and positive mental state [30]. ② Physical activity facilitates flow experiences. For instance, individual sports such as hiking, swimming, and running—which involve extended periods of rhythmic physical activity—induce the brain into a meditative state. This generates mindfulness-like psychological effects, promoting a fusion of relaxation and alertness within the brain. Consequently, it enhances a calm and balanced mental state alongside a vibrant, positive psychological disposition [31]. ③ Participation in sports enhances social support. Team sports such as frisbee and ball games create social interaction opportunities, strengthening participants’ sense of belonging, achievement, and self-confidence. This reinforces the perception of being valued and contributing to the group, promoting emotional calmness throughout the activity [32], consistent with social cognitive theory [11]. ④ Sports participation shifts attentional focus. The cognitive demands of sports require concentration, which competes for cognitive resources and effectively distracts individuals from distressing thoughts that threaten emotional stability. This attentional shift helps break cycles of anxiety and fosters emotional regulation and stability [33].

### 4.2. Robustness testing

To address potential endogeneity issues, this study employs the instrumental variables approach. First, the selection of instrumental variables requires that they be correlated with the independent variable while exhibiting exclusivity (i.e., no correlation) with the dependent variable. Second, daily stair climbing was selected as the instrumental variable. The rationale is as follows: on one hand, stair climbing and sports participation both share physical activity characteristics, satisfying the correlation requirement between the instrumental variable and the independent variable. On the other hand, stair climbing constitutes a necessary component of daily life behavior, while changes in psychological state are primarily driven by event-induced cognitive processes, fulfilling the exclusivity requirement between the instrumental variable and the dependent variable. Finally, second-order least squares regression was employed for statistical analysis. As Table 3 demonstrates, all X variables exhibit statistical significance at p < 0.001 and pass both the weak instrumental variable test (WIT) and unidentifiability test (UT). This indicates no endogeneity issues and demonstrates robust validity.

**Table 3:** Robustness test (N = 2577).

Variable	A17		E9		A17_1	
	①	②	①	②	①	②
X	.339(.054)***	.706(.148)***	.339(.054)***	.489(.117)***	.339(.054)***	.767(.145)***
CV	YES	YES	YES	YES	YES	YES
_con	2.512(4.113)	9.904(3.734)**	2.512(4.113)	10.538(2.946)***	2.512(4.113)	.120(3.642)
WIT	39.049(16.38)		39.049(16.38)		39.049(16.38)	
UT	38.628***		38.628***		38.628***	
R^2^	.876		.923		.867	

### 4.3. The impact of sports participation on the psychological state of the Chinese people under conditions of limited sports resources

As demonstrated by Model 3 in Table 4, under favorable sports environments, physical activity participation exhibits statistical significance at the p < 0.05 level for both depression and despondency (A17) and calmness and composure (E9) among the Chinese population. For vitality (E10), the significance level is p < 0.01. Observing the R² values, compared to Model 2, depression and despondency (2: R² = .081; 3: R² = .078) decreased by approximately 4%, peace of mind (2: R² = .041; 3: R² = .038) decreased by approximately 2%, and vitality (2: R² = .082; 3: R² = .082) remained consistent. It is evident that the positive correlation has weakened to some extent. Possible reasons include: ① Due to persistent imbalances and inadequacies in China’s social development, residential areas with favorable exercise environments are predominantly concentrated in densely populated regions. While these settings provide suitable conditions for physical activity participation, the high concentration of participants slightly diminishes the correlation between exercise engagement and psychological well-being under such circumstances. This finding validates the built environment deficit theory [19]. ② Better sports environments often feature beautiful scenery or well-maintained modern facilities. Such high-quality settings may enhance feelings of calmness and vitality, potentially weakening the correlation between sports participation and psychological well-being. Model 4 demonstrates that sports participation has no statistically significant effect on depression (p > 0.05) but exhibits statistical significance at the p < 0.001 level for calmness and vitality. This validates Hypothesis H4. Possible explanations include: ① Moderate environmental challenges enhance psychological resilience. Engaging in sports within a challenging environment stimulates physiological stress transformation mechanisms. The body accelerates sweating to rapidly metabolize cortisol, thereby improving negative mental health issues like depression and despondency—an effect superior to seated meditation [34]. ② It fosters a social support buffer. Team sports mitigate the impact of poor exercise environments by building emotional bonds. When trailing in scores, participants choose to communicate with teammates. This social support system effectively reduces the disparity in exercise environments and improves psychological states [35]. This to some extent undermines the applicability of the built environment deficiency theory [19].

**Table 4:** Results of the impact of sports participation on the psychological state of the Chinese people under conditions of access to sports resources.

Variable	3: Good exercise environment			4: Poor exercise environment			5: Well-equipped sports facilities		
	A17	E9	E10	A17	E9	E10	A17	E9	E10
A30_9	.131(.054)*	.112(.053)*	.157(.052)**	.026(.027)	.116(.027)***	.222(.027)***	.026(.032)	.131(.032)***	.254(.032)***
CV	YES	YES	YES	YES	YES	YES	YES	YES	YES
cut1	−29.436(9.787)	−25.849(9.650)	6.286(9.422)	10.667(5.580)	−16.088(5.553)	6.165(5.468)	−13.908(6.456)	−21.095(6.533)	9.454(6.388)
N	616	616	616	1961	1961	1961	1489	1489	1489
R^2^	.078	.038	.082	.085	.042	.081	.079	.045	.088
Variable	6: Lack of sports facilities			7: High sports safety			8: Low sports safety		
	A17	E9	E10	A17	E9	E10	A17	E9	E10
A30_9	.001(.042)	.057(.041)	.141(.041)***	.001(.033)	.133(.033)***	.236(.033)***	.081(.035)*	.092(.035)**	.181(.034)***
CV	YES	YES	YES	YES	YES	YES	YES	YES	YES
cut1	−8.114(9.124)	−7.815(8.896)	9.675(8.721)	−9.194(6.732)	−9.772(6.651)	7.258(6.619)	−22.867(7.025)	−28.348(7.009)	4.176(6.767)
N	779	779	779	1333	1333	1333	1244	1244	1244
R^2^	.091	.038	.075	.068	.035	.080	.089	.047	.075

According to Model 5, the effect of sports participation on depression among Chinese people was statistically insignificant (p > 0.05). However, its effects on calmness and vitality were both statistically significant at the p < 0.001 level. Observing the R² values, compared to Model 2, calmness (2: R² = .041; 5: R² = .045) increased by approximately 10%, while vitality (2: R² = .082; 5: R² = .088) increased by about 7%. Possible reasons include: ① Comprehensive modern sports facilities offer diverse athletic options, boosting willingness to participate in physical activities. In recent years, digital transformation has propelled rapid development across all sectors, with sports facilities experiencing exponential digitalization and intelligent upgrades. This provides personalized sports participation services and enhanced safety measures for athletes. Consequently, under conditions of well-equipped facilities, the correlation between sports participation and a calm, energetic state of mind has strengthened [36]. ② The motivational threshold for sports participation has risen. Compared to environments lacking facilities, long-term engagement in sports within well-equipped settings yields tangible physical and mental health benefits. This encourages participants to develop sustained motivation to pursue these enduring advantages [37]. Model 6 demonstrates that sports participation shows no statistically significant effect on depression and mental well-being (p > 0.05), but exhibits statistical significance at p < 0.001 for vitality. This validates Hypothesis H5. Possible reasons include: ① The lack of adequate sports facilities creates a psychological compensation effect. Damaged courts and rudimentary equipment make it difficult for participants to focus on improving movement stability, accuracy, and coordination, intensifying unpleasant experiences. This weakens the positive association between sports participation and reduced depression, frustration, and increased mental calmness [38]. ② The lack of sports facilities weakens social support. The scarcity of sports facilities creates a disruptive effect among participants, hindering the initiation of physical activity and the smooth experience during its process, thereby undermining the association between sports participation and reduced depression and increased mental calmness [20].

According to Model 7, the effect of sports participation on depression among the Chinese population was statistically insignificant (p > 0.05). However, it demonstrated statistical significance at the p < 0.001 level for calmness and vitality. Model 8 demonstrates that sports participation has a statistically significant effect on depression among the Chinese population at the p < 0.05 level. It also has a statistically significant effect on calmness at the p < 0.01 level and on vitality at the p < 0.001 level. Only under conditions of high sports safety was there no correlation between sports participation and depression or despondency; under all other conditions, a positive correlation was observed. Hypothesis H6 was thus validated. Possible reasons: ① For individuals experiencing negative mental health issues such as depression and despondency, family members, sports service providers, and community members tend to offer them special care and provide safe exercise environments. This abundance of emotional support and comprehensive special attention weakens the correlation between sports participation and depression under sports safety conditions [15]. ② Individuals with negative mental health issues such as depression activate their risk recognition and coping systems when engaging in sports participation under conditions of low sports safety. To avoid exercise risks, the subconscious mind focuses attention on the process of sports participation, thereby suppressing depressive and despondent thoughts. Simultaneously, sports participation stimulates the release of neurotransmitters like dopamine and endorphins, which regulate mood and help alleviate depression. This further strengthens the association between sports participation and depression, partially challenging perspectives from Environmental Safety Assessment Theory [22] and Health Belief Theory [23].

## 5. Research conclusions and contributions

### 5.1. Research conclusions

First, sports participation exhibits a comprehensive positive correlation with the psychological state of the Chinese people.

Second, robustness tests using the instrumental variables method indicate that the correlation between sports participation and the psychological state of the Chinese people is free from endogeneity issues and demonstrates strong robustness.

Third, the correlation between sports participation and psychological state varies significantly under different sports environments, sports facilities, and sports safety conditions: ① Under favorable sports environments, a comprehensive positive correlation exists; under poor sports environments, no correlation is found with depression and despondency. ② Under well-equipped sports facilities, a positive correlation exists with calmness and vitality; under inadequate sports facilities, no correlation is found with depression and despondency or calmness. ③ Under low sports safety conditions, a comprehensive positive correlation exists; under high sports safety conditions, no correlation is found with depression and despondency.

### 5.2. Theoretical contributions

First, examining the effects of sports participation on different psychological states, as well as the validity of the theoretical arguments supporting these effects, provides a valuable clarification of the competing theoretical perspectives and helps identify the most applicable theoretical guidance. Upon analysis: ① Perspectives grounded in neurotransmitter regulation theory [15] and social cognitive theory [11] demonstrate strong applicability. However, the applicability of flow interruption theory [13] is diminished. ② The inhibitory viewpoint based on overstress theory [16,17] has yet to be substantiated.

Second, the study incorporates the sports resources framework into the design logic of the relationship between sports participation and psychological states, revealing the validity and applicability of specific supporting arguments under varying conditions of the sports environment, facilities, and safety. Specifically, ① This provides richer theoretical support for the variable relationship between sports participation and psychological states. ② it broadens the explanatory scope and precision of social support theory [20]; ③ The built environment deficit theory [19] reveals an inherent contradiction and conflict within the variable relationships between sports participation and psychological states. The applicability of perspectives from ecological sports theory [18], environmental safety assessment theory [22], and health belief theory [23] is consequently diminished.

### 5.3. Practical implications

(1) Comprehensively enhance the positive impact of sports participation on mental well-being and vitality. As demonstrated by the statistical results above, the correlation between sports participation and mental well-being/vitality only fails to reach statistical significance at the p < 0.05 level under conditions of inadequate sports facilities. In all other conditions, the correlation exhibits statistical significance at the p < 0.05 level. To further enhance the positive impact of sports participation on the mental well-being and vitality of the Chinese people, it is recommended to organize groups including experts, physical education teachers, social sports instructors, and fitness coaches. Through public lectures, sports courses, community sports guidance, and fitness coaching, they should educate the public on the efficacy of sports participation in improving mental well-being and vitality, along with the specific physiological and biochemical mechanisms and social support principles involved. Promote nationwide sports participation to broadly enhance the psychological well-being of the Chinese population. Additionally, collaborate with higher education institutions offering sports-related majors to establish practical internship opportunities in community sports instruction for sports students. This approach will provide citizens with regular, scientifically grounded sports participation guidance while simultaneously strengthening students’ practical skills.
(2) Implement smart sports resource development to enhance the positive impact of sports participation on mental well-being. First, as demonstrated by Model 3–4, improved sports environments can comprehensively strengthen the correlation between sports participation and the mental health of the Chinese population. Therefore, it is recommended to fully implement the “15-minute fitness circle” strategy, establish forest-based sports environments, formulate plans for sports environment conservation and restoration, and enhance the moderating role of sports environments in the relationship between sports participation and the mental health of the Chinese population. Second, as demonstrated by Models 5–6, comprehensive sports facilities further strengthen the correlation between sports participation and mental well-being and vitality. Therefore, it is recommended to increase sports facility construction in densely populated areas, promote the digitalization and modernization of sports facilities, and improve diversified science popularization programs for sports facilities. This will enhance the moderating role of sports facilities in the relationship between sports participation and mental well-being and vitality. Finally, as demonstrated by Models 7–8, establish separate zones for complete sports safety and controlled sports risk. Completely safe sports safety zones should have no participation restrictions and be fully accessible to all citizens. Controlled-risk sports safety zones should impose participation conditions, such as dedicated entry points and security personnel, and require participants to exercise with familiar companions. Individuals with severe mental health issues must be accompanied by relatives or professionals. This approach strengthens the moderating effect of sports safety conditions on the relationship between sports participation and the mental well-being of the Chinese population.

### 5.4. Research limitations

This study has limitations that warrant further refinement in future research. The fixed-year data employed in this study, coupled with the inconsistent variables disclosed across different years in the CGSS, precludes further investigation into the evolution of causal relationships.
